# Remotely sensed data contribution in predicting the distribution of native Mediterranean species

**DOI:** 10.1038/s41598-025-94569-y

**Published:** 2025-04-11

**Authors:** Ahmed R. Mahmoud, Emad A. Farahat, Loutfy M. Hassan, Marwa Waseem A. Halmy

**Affiliations:** 1 Botany and Microbiology Department, Faculty of Science, Helwan University, P.O. Box: 11795, Helwan, Egypt; 2 Department of Environmental Sciences, Faculty of Science, Alexandria University, P.O. Box: 21511, Alexandria, Egypt

**Keywords:** Climate change, Conservation planning, Habitat characterization, Maxent, MODIS data, Species distribution modeling (SDM), Ecology, Plant sciences

## Abstract

The global change threats significantly alters the ecological distribution of species across different ecosystems. Species distribution models (SDMs) are considered a widely used tool for assessing the global impact on biodiversity. Recently, remote sensing data have been used in a growing number of studies to predict species distribution and improve SDMs performance. This study evaluates the contribution of spectral indices in species distribution modeling using MaxEnt. We compared models based on spectral indices data (RS-only), environmental variables (EN-only), and their combination (CM) to predict the distribution of three key Mediterranean native species: *Thymelaea hirsuta*, *Ononis vaginalis*, and *Limoniastrum monopetalum*. The combined models (CM) demonstrated superior performance with excellent accuracy measures values compared to other models. Jackknife tests revealed both environmental factors (e.g., distance to coastline, mean temperature of wettest and driest quarters) and spectral indices (e.g., NDWI, LST) contributed substantially to predicting the studied species. The findings emphasize the importance of integrating diverse data sources to improve the accuracy of SDMs, particularly in heterogeneous landscapes like the Mediterranean region. This integrated approach provides a more comprehensive understanding of species spreading patterns and is critical for effective management and conservation strategies.

**Supplementary Information:**

The online version contains supplementary material available at 10.1038/s41598-025-94569-y.

## Introduction

The accelerating pace of global change poses a significant threat to biodiversity, altering the ecological distribution of species and driving rapid rates of species extinction or expansion^1^. Global change, particularly climate change, has a profound impact on Mediterranean ecosystems, significantly altering plant distribution and biodiversity. High environmental variability among habitats of wild species affects their persistence and population dynamics^2,3^. Deciphering how plant communities and environmental factors interact is crucial for understanding patterns of communities’ structure and species composition at different scales^4^. Recognizing the pattern of the environmental variables is critical for detecting differences in plant species distribution and understanding the requirements of species for ecological restoration, management, and the development of new plantations^5^. These dynamic shifts underscore the critical need for robust tools to assess and predict the impact of these changes on biodiversity. Species distribution models (SDMs) have emerged as a pivotal tool in this endeavor, providing insights into the potential future distributions of species under various environmental change scenarios^6,7^. Species distribution models (SDMs) are recognized as models that empirically study the relationship between the geographical distribution of species or communities and their current environmental conditions^8^. SDMs have been widely employed for predicting changes in species distribution under climate change and for creating ecological suitability mapping under current and future conditions^9,10^. Using different bioclimatic, topographic, edaphic, and remote sensing indices can dependably estimate the ecological niche for the species.

In recent years, the incorporation of remote sensing (RS) data into SDMs has garnered considerable attention. Remote sensing has been one of the most powerful efficient approaches in terms of time and costs that provide observations useful for modelling distribution patterns of key species^11^. The integration of different predictor sets in SDMs approaches can strongly influence the precision and reliability of results^12,13^. The capability of using remote sensing data of different resolutions for mapping and predicting of plant species distribution has been investigated^13–16^. Spectral indices derived from remotely sensed have been utilized effectively as environmental predictors in SDMs^13,15^. The availability of multi-temporal satellite data with varied spatial resolutions plays a crucial role in modeling the distribution of wild species. This can be summarized in three main contributions: (1) it expands the range of environmental variables considered beyond just topographic and climatic parameters, (2) enhances the spatial resolution of input data by utilizing direct georeferenced measurements rather than interpolated surfaces, and (3) incorporates seasonal information from multi-temporal remote-sensing imagery, providing more detailed and dynamic insights into species distribution patterns^15,17^. Advancements in analytical techniques, increased computational capacity, improved sensor fusion and networking capabilities, and the availability of free satellite data have significantly enhanced the application of remote sensing in species distribution modeling (Turner, 2014). Remote sensing data, particularly from the Moderate Resolution Imaging Spectroradiometer (MODIS), offer a wealth of information that can enhance the accuracy of SDMs. The use of MODIS satellite data in species distribution models has been hailed as a transformative approach for providing simple and flexible distribution predictions^18,19^. The use of species distribution models (SDMs) and remote sensing technologies, such as MODIS data, is crucial for predicting these shifts and planning effective conservation strategies. By capturing detailed habitat characteristics and environmental gradients, remotely sensed data can refine predictions and identify critical areas for conservation^20^. These tools are essential for mitigating the adverse effects of global change on Mediterranean plant diversity, especially in the most vulnerable arid regions^21^.

Mediterranean ecosystems are highly sensitive to climate variations due to their unique climatic and ecological characteristics, which include hot, dry summers and mild, wet winters^22^. The Mediterranean basin is recognized as a biodiversity hotspot, housing a rich variety of plant species that are adapted to its distinct climatic conditions^23^. However, climate change exacerbates the aridity in these regions, leading to shifts in plant communities and threatening species that are unable to adapt to the rapid changes^24^. In the arid parts of the Mediterranean, such as North Africa, these impacts are even more pronounced. Increased temperatures and altered precipitation patterns can result in habitat loss and the decline of native plant species, which are often replaced by more drought-tolerant invasive species^25^. This shift in species composition not only affects the ecological balance but also impacts on the services these ecosystems provide, such as soil stabilization and water regulation^26^. Moreover, the interaction of climate change with other human-induced pressures, such as land-use change and pollution, further complicates the survival of many native Mediterranean species^12,27^. *Thymelaea hirsuta* (L.) Endl., *Ononis vaginalis* Vahl, and *Limoniastrum monopetalum* (L.) Boiss are three endemics and key indicators of the main habitats of the Mediterranean coastal deserts of Egypt. The three endemic species are threatened by the reduction in their populations as well as the exponential loss and degradation of their natural habitats brought on by recent human activities^12,13^. This study aims to explore the contribution of remotely sensed data in predicting the distribution of the three native Mediterranean plant species. The predictive performance of different modelling techniques in determining the geographical distribution of the three native Mediterranean species will be compared utilizing a combination of environmental variables and remotely sensed data. The outcomes of these models can help refine conservation planning and management strategies, ensuring a more comprehensive and effective approach in safeguarding these Mediterranean native species amidst changing environmental conditions.

## Materials and methods

### Study area and studied species

**Fig. 1:**
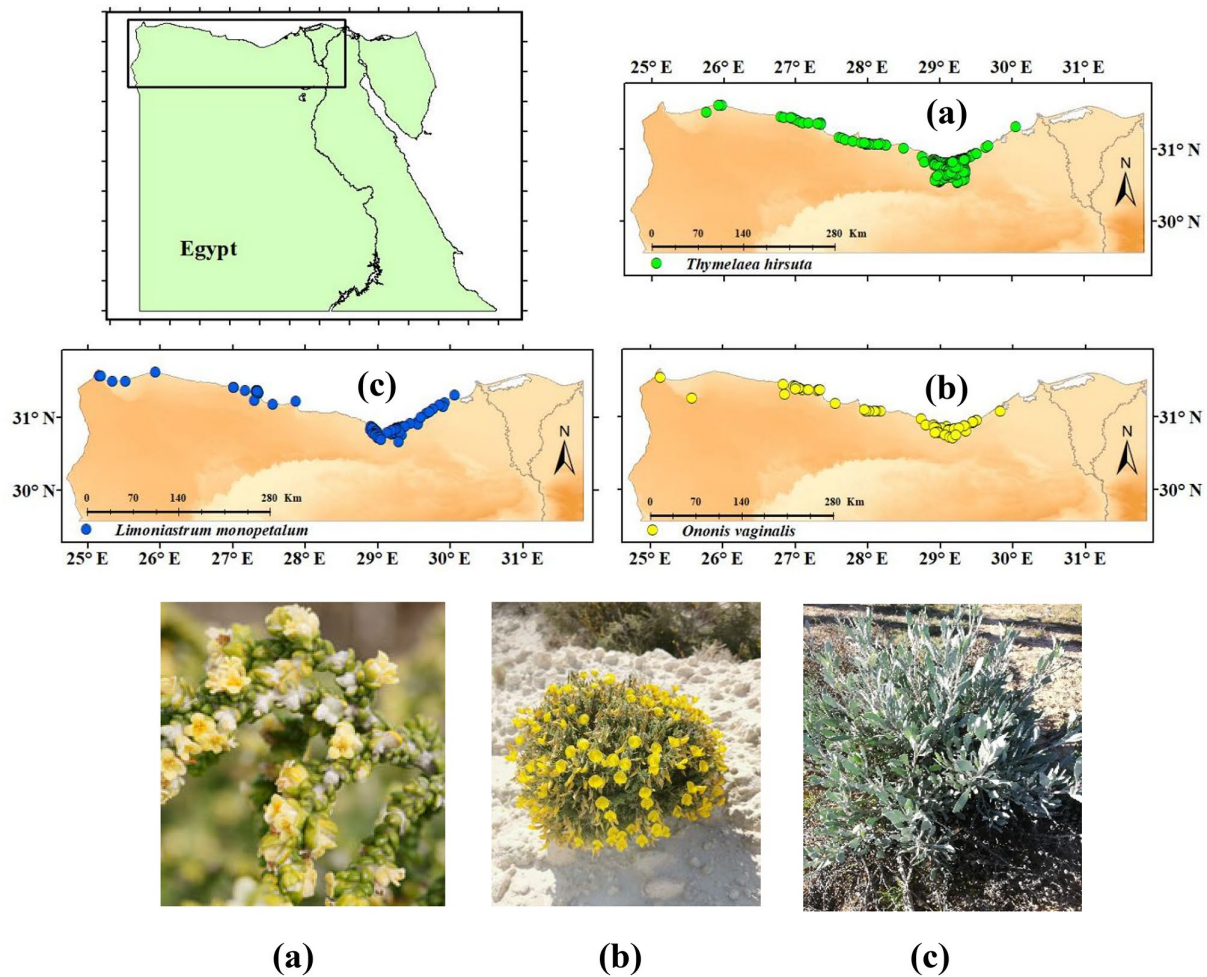
Study area surveyed for the occurrence of the studied species indicating locations of the collected occurrence records and pictures of the studied species (**a**) *Thymelaea hirsuta* (green circle), (**b**) *Ononis vaginalis* (yellow circle) and (**c**) *Limoniastrum monopetalum* (blue circle) collected through field surveys. The maps in figure were produced by the authors within the framework of the GIS software package ArcMap 10.2 (ESRI, 2013). The photographs were taken by the authors.

The current study focused mainly on the western Mediterranean coastal region in Egypt, which extends between 24°42′36″ and 31°51′0″E, and between 29°34′12″ and 31°40′12″N (Fig. 1). The total study area was represented by 175,018 km^2^. The region represents the northern coast of the Western Desert, which extends as a thin belt of land parallel to the Mediterranean Sea that narrows or widens according to the position of the Western Desert Plateau that outlines its southern boundary. It has an average north-south width of 20 km running from sea landward and is bordered by Lake Mariut on the east side. Climatically, the western Mediterranean coastal land belongs to the dry arid climatic zone of Koppen’s classification system and the Mediterranean bioclimatic zone^28–30^. Generally, it is considered the least arid belt of Egypt, with a mean annual precipitation that ranges between 100 and150 mm/year. The annual mean maximum temperature ranges from 25.3 °C to 23.8 °C, while the annual mean minimum temperature ranges from 13.3 °C to 15.1°C^31^.

The three studied species are endemics and key indicators species of the main habitats of the northwestern coastal deserts of Egypt. *Thymelaea hirsuta* (L.) Endl. (Family Thymelaeaceae), *Ononis vaginalis* Vahl (Family Fabaceae), and *Limoniastrum monopetalum* (L.) Boiss (Family Plumbaginaceae). The three endemic species are threatened by the reduction in their populations as well as the exponential loss and degradation of their natural habitats brought on by recent human activities^12,13^.

***Thymelaea hirsuta*** **(L.) Endl.** (Family Thymelaeaceae) is a native species of North Africa. *T. hirsuta* is commonly known as “Methnane”, is considered as one of the most widely distributed species along the relatively rainy Mediterranean coastal desert and extends southwardly to approximately 70 km from the shoreline. The species cannot be found beyond 90 km south of the coast. It can be found in the different sections of the Mediterranean phytogeographical region. Historically, *T. hirsuta* has been recognized as an important medicinal plant and more research has been carried out on the medical applications of the species. For instance, El Amrani et al. reported that *T. hirsuta* possesses both hypoglycaemic and antidiabetic activities^32^. Amari et al. indicated that *T. hirsuta* extracts are rich sources of natural antioxidants with a potential for used as alternative to synthetic antioxidants^33^. Felhi et al. detected the anti-microbial activity exhibited by the essential oil isolated from *T. hirsuta*^34^. Dahamna et al. reported that extracts of *T. hirsuta* possess antifungal activity^35^.

***Ononis vaginalis*** **Vahl** (Family Fabaceae) is an evergreen subshrub that grows primarily in the subtropical biome. The species inhabits the coastal dunes habitat along the western Mediterranean littoral of Egypt^36^. The distribution of *O. vaginalis* is restricted to the coastal dunes due to the rainfall rate (150 mm/year), while it diminishes in the inland desert^37^. Mahmoud et al. reported that *O. vaginalis* contain high crude protein and moderate fiber content, along with high in vitro dry matter digestibility^38^. Amer et al. isolated many flavonoids such as apigenin, chrysin, eupatilin, and astragalin from *O. vaginalis*^39^.

***Limoniastrum monopetalum*** **(L.) Boiss** (Family Plumbaginaceae) is a native species to the salt marshes and coastal dunes at the northern coast of Egypt and other Mediterranean nations^40^. *L. monopetalum* adapts to many environmental stresses such as salinity, intense radiation, water deficiency, high temperatures, and nutrients deficient soils, which makes *L. monopetalum* an ideal potential ornamental species for xeric landscaping in arid Mediterranean landscapes^41,42^. *L. monopetalum* can be used in the stabilization of coastal dunes as it has been reported as a good sand accumulator, windbreaker^41^, an inhibitor of soil erosion, and tolerant of oil-contaminated soils^43^. It has potential for use in phytoremediation of sites polluted by heavy metals and petroleum hydrocarbon^44^. *L. monopetalum* is rich in nutritive content and its vegetative parts can be used as raw material for fodder production^45^. Moreover, *L. monopetalum* has high contents of phenolics, and consequently, it is considered as a natural source of antioxidants for human consumption. Mohammed et al. found that the aqueous extract of *L. monopetalum* has a good ability to produce silver nanoparticles^46^.

Experimental research and field studies on plants, including the collection of plant material, comply with relevant institutional, national, and international guidelines and legislation.

Identification of the plant material used in the study was performed by Prof. Dr./ Loutfy Mohsen Hassan professor of plant ecology and flora at Faculty of Science- Helwan University – Botany and Microbiology department. Some of collected samples was kept in Helwan University Herbarium (HEU) (vouchers no 0111064 to 0111079 five vouchers for each species).

### Species occurrence data collection

The three species geographic information was obtained through extensive fieldwork in the western Mediterranean coastal region in Egypt. We obtained 449 geographic record data of the three endemic species through field surveys (Fig. 1). *T. hirsuta* was the most geographic record obtained by 310 records (69% of the total points) while 65 occurrence points (14.5% of the total) were *O. vaginalis* and 74 occurrence points (15.5% of the total) were *L. monopetalum*. The field surveys covered the main habitats in the western Mediterranean coastal region, where plots were selected randomly to represent the major physiographic variations. The locations of the occurrence points of the plant species studied were recorded using a global positioning system (Garmin GPSMAP 64sx GPS). The coordinates of these points were included in the geographic information system (GIS) domain and made ready for use in the subsequent analysis.

### Environmental data selection

Environmental variables are measurable factors that characterize the physical, chemical, and biological conditions of a specific environment. These variables significantly influence the distribution, behavior, and survival of organisms and can include climatic factors (e.g., temperature, precipitation), topographic features (e.g., elevation, slope), soil properties, and other ecological parameters^47^.

Guided by the conceptual framework for the environmental factors of importance in plant species distribution models by the environmental dataset used encompassed a broader range of factors^48^. The variables used in the study are categorized as 23 were bioclimatic variables which were downloaded from the WorldClim dataset version 2.1 at 30 arc-seconds (1 km) spatial resolution averaged for the years 1970–2000^49^. The elevation data were used to represent the topographic features in the study area. Elevation data were downloaded from the United States Geological Survey Dataset (https://www.usgs.gov^50^. Nine soil variables from the SoilGrids dataset (https://soilgrids.org) were used to represent the edaphic conditions of the study area^51^. The soil data were obtained at 250 m spatial resolution; therefore, the data layers were resampled to match the spatial resolution of the other environmental variables layers. Habitat-type data were created based on the habitat classification scheme of Egypt^52^. To account for maritime influences due to proximity to the Mediterranean Sea, the distance to the coast was also included as a factor (Table 1).

**Table 1:** Environmental variables used in the study (sources: http://worldclim.org/version2; https://www.usgs.gov; https://soilgrids.org, and Https://earthexplorer.usgs.gov)

Abbreviation	Variables	Units
Climatic/bioclimatic variables		
bio1	Annual mean temperature	°C
bio2	Mean diurnal range (mean of monthly (max temp – min temp))	°C
bio3	Isothermality (P2/P7) × 100	
bio4	Temperature seasonality (standard deviation × 100)	
bio5	Max temperature of warmest Month	°C
bio6	Min temperature of coldest month	°C
bio7	Temperature annual range (P5–P6)	°C
bio8	Mean temperature of wettest quarter	°C
bio9	Mean temperature of driest quarter	°C
bio10	Mean temperature of warmest quarter	°C
bio11	Mean temperature of coldest quarter	°C
bio12	Annual precipitation	mm
bio13	Precipitation of wettest month	mm
bio14	Precipitation of driest month	mm
bio15	Precipitation of seasonality (coefficient of variation)	
bio16	Precipitation of wettest quarter	mm
bio17	Precipitation of driest quarter	mm
bio18	Precipitation of warmest quarter	mm
bio19	Precipitation of coldest quarter	mm
Prec	Precipitation	mm
tmax	Maximum temperature	°C
tmin	Minimum temperature	°C
tavg	Average temperature	°C
Topographic variables		
Alt	Elevation	m
Soil variables		
Bulk density	Bulk density	cg/cm^3^
Clay	Clay content	g/kg
Coarse fragments	Coarse fragments	cm^3^/dm^3^
Nitrogen	Nitrogen	g/kg
Sand	Sand	g/kg

Abbreviation	Variables	Units
Soil variables		
Silt	Silt	g/kg
Carbon density	Organic carbon density	g/dm^3^
Cation exchange	Cation exchange capacity (at pH 7)	mmol(c)/kg
pH	Soil pH	pH × 10
Habitat		
Habitat	Habitat types	–
Proximity to sea	Distance to coastline	km
Remotely sensed		
NDVI	Normalized Difference Vegetation Index	–
NDWI	Normalized Difference Water Index	–
NDDI	Normalized Difference Drought Index	–
EVI	Enhanced Vegetation Index	–
LST (Day)	Land Surface Temperature/Emissivity (Day algorithm)	K
LST (Night)	Land Surface Temperature/Emissivity (Night algorithm)	K

### Remote sensing data and spectral indices

Remotely sensed data refers to information collected about an object, area, or phenomenon from a distance, typically using satellite sensors, aerial imagery, or drone technology^53^. This data is acquired without direct physical contact and can include measurements of electromagnetic radiation (such as visible light, infrared, or microwave signals) reflected or emitted from the Earth’s surface^54^. It is widely used in environmental monitoring, land cover classification, climate studies, and geographic analysis^55^.

The capability of using remote sensing data of different resolution for mapping and predicting of plant species distribution has been investigated^14–16,56^. While remotely sensed data include both raw data and processed spectral measurements collected by remote sensors; the spectral indices are derived variables calculated using mathematical formulas applied to spectral band data^57^. In the present study the term ‘remotely sensed data’ was used to provides a more comprehensive and inclusive term that accurately represents both spectral and non-spectral remote sensing-derived datasets. To assess the contribution of remotely sensed data and spectral indices in the prediction of the distribution of the selected species, the dataset included not only spectral indices (e.g., NDVI) but also other remotely sensed variables such as Land Surface Temperature (LST) and Emissivity (LST & E). Three spectral indices derived from remotely sensed MODIS satellite data (NDVI, NDWI and NDDI) in addition to the monthly land surface temperature land surface temperature and emissivity data were used in SDMs of the studied species. The remote sensing MODIS data were downloaded from https://earthexplorer.usgs.gov^50^. The MODIS monthly NDVI (Normalized Difference Vegetation index) and EVI (Enhanced Vegetation Index) data were downloaded at 1 km (MOD13A3) V6.1. The monthly Land Surface Temperature land surface temperature (LST) and Emissivity (LST & emissivity (E) data derived from the MOD11C3 Version 6 available in global coverage at 0.05-degree (1 km) resolution were obtained for the study area. The Normalized Difference Water Index (NDWI) was computed according to Vogelmann et al.^58^ using the near infrared (NIR—MODIS band 2) and the short-wave infrared (SWIR—MODIS band 7) reflectance bands of (MOD13A3) V6.1 as follows:

$$\text{NDWI}=\frac{\text{N}\text{I}\text{R}\left(\text{M}\text{O}\text{D}\text{I}\text{S}\:\text{b}\text{a}\text{n}\text{d}\:2\right)-\text{S}\text{W}\text{I}\text{R}\left(\text{M}\text{O}\text{D}\text{I}\text{S}\:\text{b}\text{a}\text{n}\text{d}\:7\right)}{\text{N}\text{I}\text{R}\left(\text{M}\text{O}\text{D}\text{I}\text{S}\:\text{b}\text{a}\text{n}\text{d}\:2\right)+\text{S}\text{W}\text{I}\text{R}\left(\text{M}\text{O}\text{D}\text{I}\text{S}\:\text{b}\text{a}\text{n}\text{d}\:7\right)}$$

Normalized Difference Drought Index (NDDI) was calculated from NDVI and NDWI according to Omernik^59^ using the following equation.

$$\text{NDDI}=\frac{\text{N}\text{D}\text{V}\text{I}-\text{N}\text{D}\text{W}\text{I}}{\text{N}\text{D}\text{V}\text{I}+\text{N}\text{D}\text{W}\text{I}}$$

The mean of the monthly values for each spectral indices for the period from 1/1/2021 to 1/1/2022 was calculated and used in modelling the distribution of the studied species.

Finally, all these data layers representing the environmental variables were maintained at 30 arc-second (~ 1 km) resolutions and were cropped to the spatial extent of the study area using Arc GIS software v. 10.2.

### Ecological niche modelling and evaluation

Preparative exploratory data analyses, including a normality test, correlation analysis, and multicollinearity statistics, were carried out to examine the relevance of the environmental attributes. To detect multicollinearity and identify the influential variables to be used for the prediction of the studied species distribution, variance inflation factors (VIFs) were calculated. Variables with a VIF value greater than 5 were neglected as their contributions were negligible^60^. VIF was implemented using “usdm” package^61^. The distribution of each species was modelled under current climate conditions with a reduced set of variables of the initial set of variables (Table 2) after accounting for the collinearity by applying Variance Inflation Factor analysis.

**Table 2:** Selected environmental variables used for modelling, correlated variables with variance inflation factor (VIF) values > 5, and correlation threshold of 0.75 were removed to avoid problems related to collinearity.

Species	Variables	Abbreviations	VIF
*Thymelaea hirsuta*	Annual mean temperature	bio1	2.56
	Precipitation of coldest quarter	bio19	1.36
	Mean temperature of wettest quarter	bio8	2.68
	Mean temperature of driest quarter	bio9	1.67
	Elevation	Alt	3.64
	Maximum temperature	t_max_	2.57
	Habitat types	Habitat type	1.90
	Proximity to sea	Distance to coastline	3.90
*Ononis vaginalis*	Soil pH	pH	3.10
	Precipitation of coldest quarter	bio19	1.44
	Isothermality (P2/P7) × 100	bio3	1.87
	Mean temperature of wettest quarter	bio8	3.50
	Mean temperature of driest quarter	bio9	2.75
	Elevation	Alt	2.23
	Precipitation	Prec	4.50
	Habitat types	Habitat type	3.60
	Proximity to sea	Distance to coastline	2.45
*Limoniastrum monopetalum*	Precipitation of warmest quarter	bio18	2.32
	Precipitation of coldest quarter	bio19	3.68
	Mean temperature of driest quarter	bio9	1.79
	Clay content	Clay	4.42
	Coarse fragments	Coarse fragment	3.58
	Nitrogen	nitrogen	2.26
	Silt	Silt	3.24
	Elevation	Alt	2.18
	Habitat types	Habitat type	1.40
	Proximity to sea	Distance to coastline	2.35

The maximum entropy (Maxent) was applied for modelling the distribution of the studied species using Maxent version 3.4.4^62^. The models built for each species were by using remote sensing data only (RS-only), Environmental variables data only (EN-only) and combined model (CM). The models built for each species were calibrated using 70% of the occurrence records that were selected randomly. The models were run five times, and the average probability of occurrence was obtained^63^. The retained 30% of the occurrence records were then utilized for accuracy assessment and evaluation of models’ performance. To evaluate the resulting models, several measures of accuracy assessment were estimated including the overall accuracy, sensitivity, specificity, and true skill statistics (TSS). In addition, the Area Under the Curve (AUC) of the Receiver Operating characteristic Curve (ROC) was estimated to evaluate the accuracy of the resulting models. The value of the AUC closer to 1 demonstrates superior model performance. The TSS is better than AUC as it is threshold-dependent and accounts for both sensitivity and specificity, with values ranging from 0 to 1. The Jackknife test in the model was used to calculate the contribution rate of each environmental factor to the species distribution and detect the main environmental variables influencing it^62^.

### Geospatial analysis of the species’ potential distribution

The prediction results of the established models represent the occurrence probability for the species, with a range between 0 and 1, where 0 indicates the unsuitable area for the species and 1 indicates the optimal area for the occurrence of the modeled species.

**Fig. 2:**
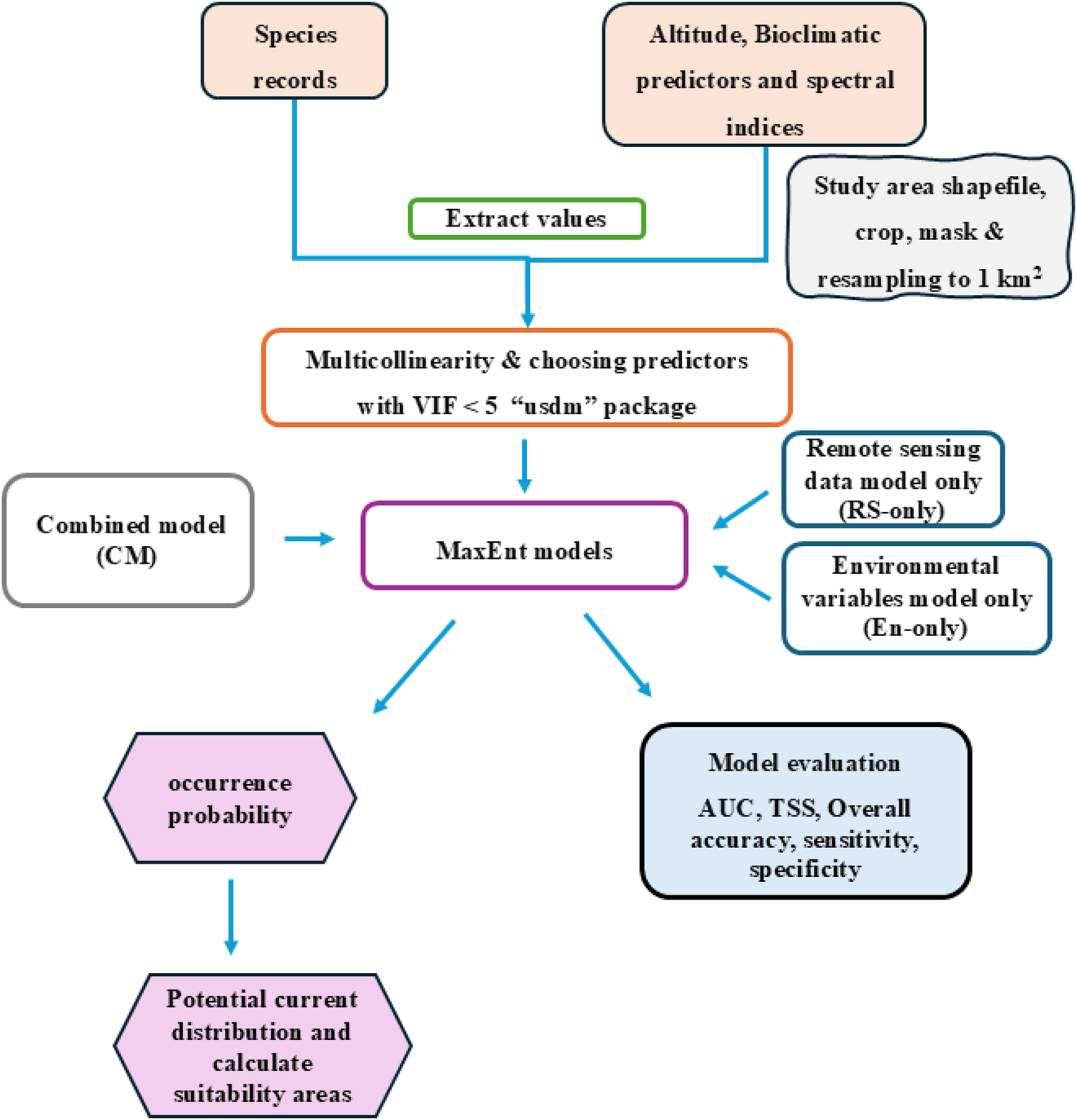
Flowchart depicting the spatial analysis and statistical processing steps used in the modeling workflow.

Additionally, to classify the study area in terms of its suitability for each of the studied species, the probability maps produced as outputs of the established models were classified based on the range of the probability of occurrence into four levels of habitat suitability into 4 classes (Table 3). The reclassification function in the Spatial Analyst Tools within the framework of ArcGIS 10.2 (Environmental Systems Research Institute 2013) was used for conducting the classification. All methodological steps are illustrated in the flowchart presented in Fig. 2.

**Table 3:** Habitat suitability classes and the range of probability of occurrence for the studied species based on environmental variables.

Species	Suitability class			
	Unsuitable	Low	Moderate	High
*Thymelaea hirsuta*	< 0.16	0.161–0.30	0.301–0.60	> 0.601
*Ononis vaginalis*	< 0.10	0.101–0.35	0.351–0.65	> 0.651
*Limoniastrum monopetalum*	< 0.10	0.101–0.35	0.351–0.65	> 0.651

## Results

### Models’ performance and environmental predictors

Overall, the combined models of the three species obtained a higher performance and predictive ability (Table 4). Average values for *T. hirsuta* of CM (Overall accuracy: 98, sensitivity: 99, specificity: 98, TSS: 0.97, AUC: 0.99), in comparison to EN-only model (Overall accuracy: 97, sensitivity: 98, specificity: 97, TSS: 0.95, AUC: 0.98) and RS-only model (Overall accuracy: 83, sensitivity: 93, specificity: 83, TSS: 0.75, AUC: 0.93). While CM for *O. vaginalis* species has average values of TSS: 0.90 and AUC: 0.985 which indicate higher performance and predictive ability than the other two models, EN-only model (TSS: 0.85 and AUC: 0.980) and RS-only model (TSS: 0.78 and AUC: 0.95). The third species *L. monopetalum* has the same results that indicate the difference in performance of the three models but with average values equal (TSS: 0.95 and AUC: 0.99), (TSS: 0.94 and AUC: 0.98) and (TSS: 0.72 and AUC: 0.93) for CM, EN-only model, and RS-only model respectively. When comparing performance metrics, it is also important to consider their scale of variation; while TSS vary between − 1 and 1 (with better models nearing one), AUC varies between 0 and 1 (also with better models nearing one).

**Table 4:** Accuracy measures and variable contributions in models predicting the potential distribution of the *s*tudied species.

Species	Model	Top variables by contribution (%)	Overall accuracy (%)	Sensitivity (%)	Specificity (%)	TSS	AUC
*Thymelaea hirsuta*	Remote sensing-only model	LST (day) (39.8)	83 ± 0.007	93 ± 0.02	83 ± 0.007	0.75 ± 0.03	0.93 ± 0.001
		NDWI (32.6)					
		NDVI (19.8)					
		EVI (6.1)					
	Environmental-only model	Distance to coastline (50.11)	97 ± 0.000	99 ± 0.006	97 ± 0.000	0.95 ± 0.006	0.98 ± 0.001
		bio9 (18.52)					
		bio8 (17.26)					
		bio1 (6.88)					
		Habitat type (4.72)					
	Combined model	Distance to coastline (46.8)	98 ± 0.001	99 ± 0.01	98 ± 0.001	0.97 ± 0.01	0.99 ± 0.001
		bio8 (21.4)					
		bio9 (12.1)					
		NDWI (6.5)					
		LST (day) (3.9)					
		t_max_ (3.6)					
		bio1 (2.4)					
*Ononis vaginalis*	Remote sensing-only model	NDWI (54.2)	92 ± 0.01	86 ± 0.10	92 ± 0.01	0.78 ± 0.09	0.95 ± 0.01
		NDVI (26.7)					
		LST (day) (16.0)					
		EVI (2.5)					
	Environmental-only model	Distance to coastline (84.04)	97 ± 0. 00	88 ± 0.13	97 ± 0.00	0.85 ± 0.12	0.980 ± 0.01
		Habitat type (9.11)					
		bio9 (3.08)					
	Combined model	Distance to coastline (85.6)	96 ± 0.01	92 ± 0.05	98 ± 0.00	0.90 ± 0.05	0.985 ± 0.01
		NDVI (3.2)					
		bio8 (2.4)					
		bio9 (2.1)					
*Limoniastrum monopetalum*	Remote sensing-only model	NDWI (53.5)	96 ± 0.008	76 ± 0.08	96 ± 0.008	0.72 ± 0.07	0.93 ± 0.001
		NDVI (27.3)					
		LST (day) (16.6)					
		LST (night) (2)					
	Environmental-only model	Distance to coastline (82.54)	96 ± 0.003	98 ± 0.034	96 ± 0.003	0.94 ± 0.036	0.98 ± 0.001
		Habitat type (10.40)					
		bio19 (2.54)					
	Combined model	Distance to coastline (84.4)	97 ± 0.002	98 ± 0.04	97 ± 0.002	0.95 ± 0.03	0.99 ± 0.001
		bio19 (3.7)					
		bio18 (2.9)					
		NDVI (2.2)					

The results of the Jackknife test of The combined prediction of *T. hirsutta* model showed that the most contributed variables were distance to coastline, Mean temperature of wettest quarter (bio8), Mean temperature of driest quarter (bio9), NDWI, LST (night), t_max_ and Annual mean temperature (bio1) with contribution percent 46.8, 21.4, 12.1, 6.5, 3.9, 3.6 and 2.4% respectively while EN-only model were distance to coastline 50.11%, followed by, bio9, bio8, bio1 and Habitat type with a relative contribution of 18.25, 17.26, 6.88 and 4.72% respectively. The RS-only model indicated that LST (Day) attained the highest contribution percentage of 39.8%, followed by NDWI (32.6%), NDVI (19.8%), and EVI (6.1%) (Table 2).

The results of the Jackknife test of The CM of *O. vaginalis* showed that distance to coastline (85.6%), NDVI (3.2%), bio8 (2.4%) and bio9 (2.4%) are the most important factors controlling the potential distribution of *O. vaginalis*. The EN-only model revealed that the distance to coastline, habitat type, and bio9 were the most important predictors of *O. vaginalis* distribution. The three predictors showed contribution values of 84.04, 9.11 and 3.08% respectively while the most important and contributing variables which influence the geographical distribution of *O. vaginalis* based on RS-only model were NDWI with 54.2%, followed by NDVI, LST (Day) and EVI that attained 26.7%, 16% and 2.5% contribution percent respectively (Table 4).

The highest mean percentage of contributions were attained by the distance to the coastline, precipitation of coldest quarter (bio19), precipitation of warmest quarter (bio18) and NDVI with contribution percent of 84.4%, 3.7, 2.9 and 2.2 respectively based on CM of *L. monopetalum* but Distance to coastline (82.54%), habitat type (10.40%), bio19 (2.54%) are the most contributed and controlling variables based on EN-only model while RS-only model revealed that the NDWI, NDVI, LST (Day) and LST (Night) were the most important variables in predicting the distribution of *L. monopetalum*. These predictors provided a relatively higher contribution in the model (53.5, 27.3, 16.6 and 2% respectively) (Table 4).

**Fig. 3:**
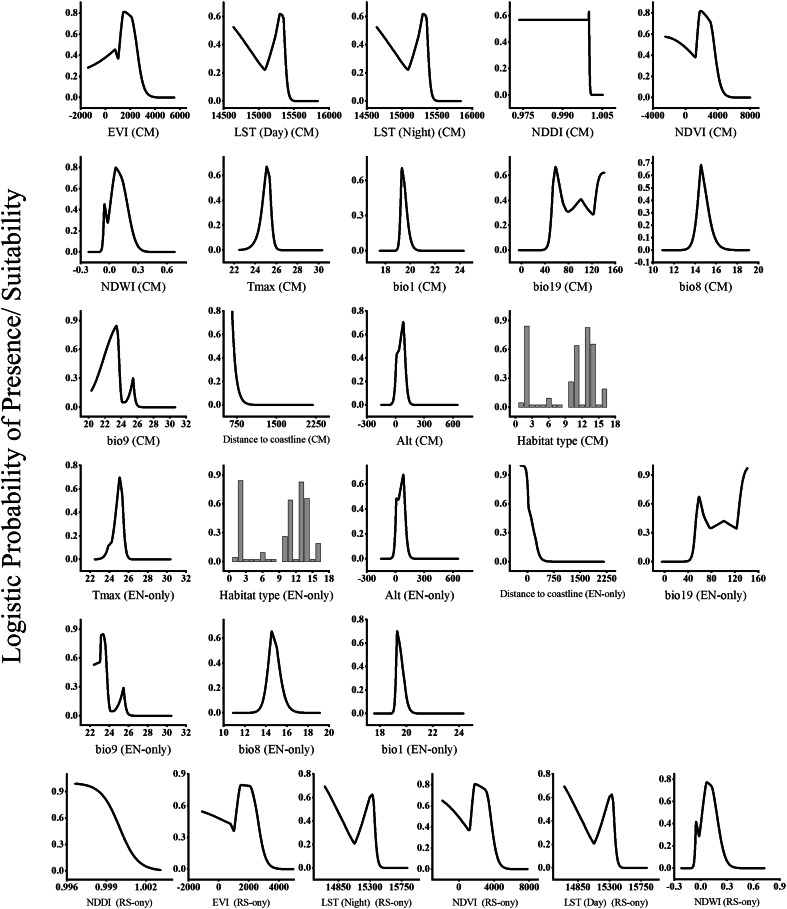
Response curves of important predictors for* Thymelaea hirsuta* species distribution model by Maxent using CM, EN-only and RS-only models.

The response curves (Fig. 3) showed the relationship between remote sensing indices and the logistic prediction of habitat suitability. The response curves of the spectral indices showed that the suitable range of the LST(Night) for the distribution of *T. hirsuta* was 292.76–309.66, whereas that for the LST (Day) ranged from 293.08 to 309.04. The range for NDVI varied from − 0.1896 to 0.5449, whereas that for NDWI varied from − 0.0952 to 0.392, the range for NDDI varied from 0.997 to 1.0025 and the range of EVI varied from − 0.1129 to 0.5332. The probability of the occurrence of the species exhibited a positive relationship with all the spectral indices except for the NDDI.

The response curves (Fig. 3) demonstrate the relationship between the probability of occurrence for *T. hirsuta* and each of the environmental variables used to create the Maxent distribution model. The curves show the range of suitability for the environmental variable used to create the Maxent distribution model of *T. hirsuta* independent of other variables. The probability of occurrence of *T. hirsuta* follows a range of the mean temperature of wettest quarter between 10 and 18 °C with an optimum value of 14.0 °C at which the species attained high probability of occurrence. The probability of the occurrence of *T. hirsuta* follows an optimum range for the annual mean temperature between 18.0 and 24.0 °C with a high probability of occurrence in areas that have an annual mean temperature of 20 °C. For mean temperature of driest quarter, and t_max_ the range of suitability was 22.37–26.18 °C and 22.45–26.15 °C-respectively. The probability of *T. hirsuta* occurrence increased in areas with precipitation of coldest quarter exceeding 36.0 mm/year. The species prefers coastal sand dunes, sandy shoreline and sand bars habitat type that are close to the coast with suitable distance from the coastline ranging from 190 to 590 m. Furthermore, the suitable elevation for the species ranged from 0 to 214.0 m with an optimal elevation of 80.0 m.

The response curves of the model based on integrating the remotely sensed data with environmental factors (Fig. 3) revealed that probability of occurrence of the species exhibited a negative relationship with the distance to coastline and the NDDI, while exhibiting a direct relationship with the rest of the variables. The optimum range of the distance to the coastline for the occurrence of the species was 0.19–0.498 km, with the suitable elevation was up to 79.41 m, the mean temperature of wettest quarter was 13.12–16.96 °C, the mean temperature of driest quarter suitable range was 20.32–26.08 °C, the annual mean temperature range was 18.90–20.53 °C, maximum temperature (t_max_) 22.45–26.12 °C, the precipitation of coldest quarter 39.89–141.2 mm, the LST (Night) 292.76–309.66, the LST (Day) 292.76–309.66, the NDVI − 0.2459–0.5413, the NDWI index − 0.090–0.367, NDDI 0.999–1.0077 and the EVI − 0.1414–0.3926. The most suitable habitat types were the coastal sand dunes, sandy shoreline, and sand bars habitats﻿.

**Fig. 4:**
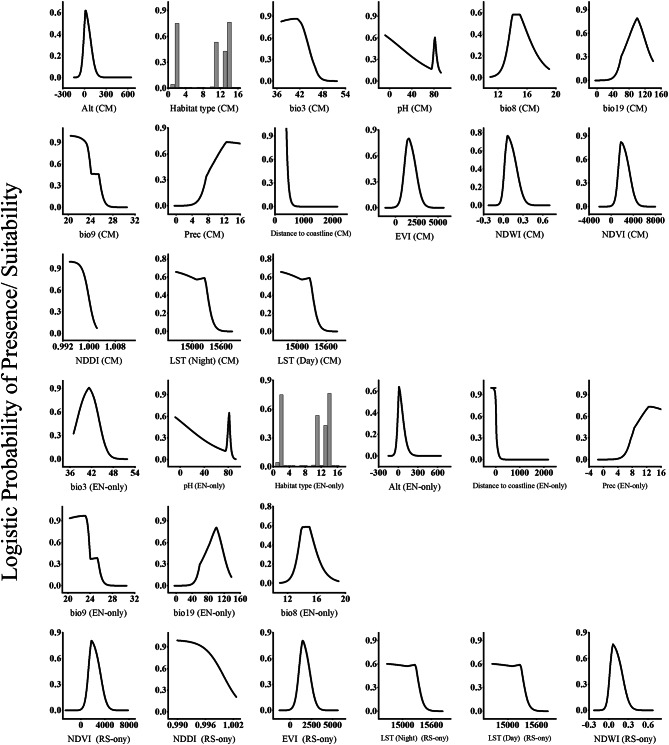
Response curves of important predictors for *Ononis vaginalis* species distribution model by Maxent using CM, EN-only and RS-only models.

The response curves showed the relationship between the spectral indices and the probability of occurrence of (Fig. 4). According to the response curves of the NDWI, NDWI NDVI and EVI showed a positive relationship with the probability of occurrence of *O. vaginalis*. The suitability range of the NDWI, NDVI and EVI for the occurrence of the species was − 0.10 to 0.441, − 0.0183 to 0.5354 and − 0.0244 to 0.3926, respectively. In contrast, LST (Night), LST (Day) and NDDI showed a negative relationship with the probability of occurrence of *O. vaginalis*. The suitability range of LST (Night), LST (Day) and NDDI for the occurrence of the species was 305.06 to 312.46, 305.06 to 312.46 and 0.995 to 1.0026, respectively.

The response curves (Fig. 4) were generated to demonstrate the relationship between the probability of occurrence for *O. vaginalis* and each of the environmental variables used to create the Maxent distribution model. The curves show the range of suitability for the environmental variable used to create the Maxent distribution model of *O. vaginalis* independent of other variables. Except for the annual precipitation (bio9) and distance to the coastline, the response curves of all the remaining variables revealed a positive nonlinear relationship with the probability of *O. vaginalis* occurrence. High probability of *O. vaginalis* occurrence was predicted when the mean temperature of wettest quarter (bio8) ranged from 10.9 to 19.04 °C. The optimum value of the isothermality (bio3) for the occurrence of *O. vaginalis* ranged from 36.9 to 51.8 °C. The optimum range of the precipitation of coldest quarter (bio19) was 17.23 to 137.9 mm per year, while that of the annual precipitation ranged from 3.16 to 17.6 mm per year. The most suitable elevation was 9.55 m, while the suitable soil pH range was from 7.4 to 9.2. The suitable habitat for *O. vaginalis* were the coastal sand dunes, sandy shoreline, and sand bars. The probability of *O. vaginalis* occurrence exhibited a negative relationship with the annual precipitation (bio9) and the distance to the coastline.

The response curves of the predictors used in the model that integrated the remotely sensed data with environmental factors show the relationship between these predictors and the probability of occurrence of *O. vaginalis* (Fig. 4). The suitable ranges for the main contributing variables to the prediction of the species as was revealed from the response curve were as follows: the mean temperature of wettest quarter (bio8) ranged from 11.4 to 15.01 °C, the isothermality (bio3) was 40.9–48.38 °C, whereas the mean temperature of driest quarter (bio9) was 20.3–27.53 °C. On the other hand, the precipitation of coldest quarter (bio19) was 30.6–140.1 mm per year, while the suitable habitat occurs also when the precipitation seasonality ranged from 3.82 to 12.55 mm per year. The suitable elevation range for *O. vaginalis* was − 0.0–224.0 m. Suitable habitat type was the coastal sand dunes. The suitable soil pH for *O. vaginalis* was 7.486 to 9.24, and the suitable distance from the coast ranged from 0.08 to 0.26 km. The optimum range for the spectral indices was 305.06–312.46 for the (LST (Night)), 293.012–312.62 (LST (Day)), 0.0033–0.5365 for the NDVI, − 0.087–0.423 for NDWI, 0.995–1.0023 for NDDI, and − 0.0070–0.3934 1for EVI. The precipitation, bio19, elevation, EVI, NDVI and NDWI exhibited a positive relationship with the probability of occurrence of *O. vaginalis*.

**Fig. 5:**
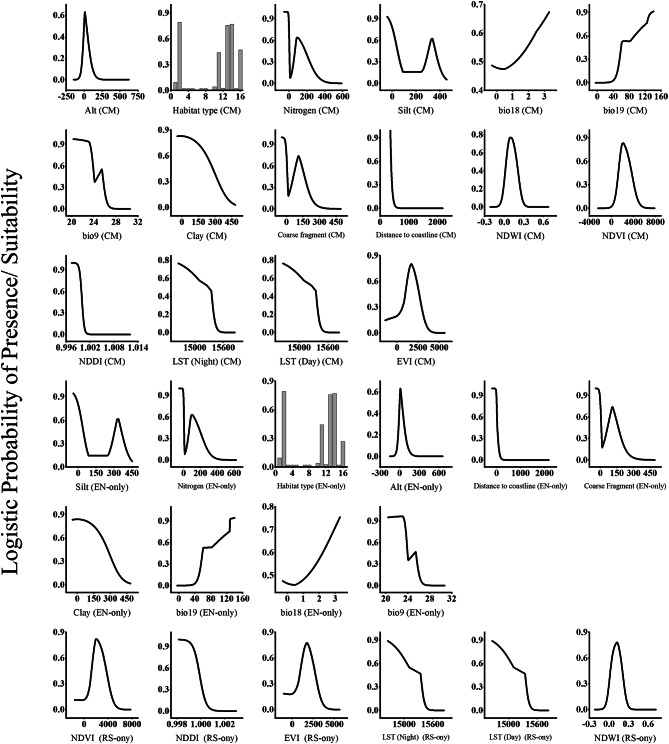
Response curves of important predictors for *Limoniastrum monopetalum* species distribution model by Maxent using CM, EN-only and RS-only model.

The response curves of the model based on the remotely sensed data alone showed the relationship between the spectral indices and the probability of occurrence of *L. monopetalum* (Fig. 5), where the suitable range of the LST (Night) was 293.12 to 310.52, LST (Day) was from 293.12 to 310.52, the NDDI varied from 0.998 to 1.001. These indices showed a negative relationship with the probability of the occurrence of *L. monopetalum.* In contrast, NDVI, NDWI and EVI showed a positive relationship with the probability of occurrence of *L. monopetalum*. The suitable range of NDVI varied from 0.0071 to 0.55, whereas the NDWI varied from − 0.103 to 0.343, and the EVI varied from 0.005 to 0.4289.

It is apparent that the mean temperature of the driest quarter (bio9) ranged from 20.3 to 26.72 °C with a peak at 25.4 °C contributing positively to the distribution of species (Fig. 5). On the other hand, the distribution of *L. monopetalum* responds positively with the increase in precipitation of coldest quarter (bio19) and precipitation of warmest quarter (bio18). The most suitable elevation for *L. monopetalum* distribution was 9.2 m. It seems that the most suitable habitat for *L. monopetalum* is coastal sand dunes and sandy shorelines, salt marshes, and sand bars. Soil clay fraction has negative influence on the distribution of *L. monopetalum*, while coarse fragment and silt fraction showed an optimum peak at 108.95 and 335.7. The suitable distance to coastline for *L. monopetalum* ranged from 0.07 to 0.292 km.

The response curves of the predictors used in the model integrated the remotely sensed data with environmental factors (Fig. 5) show the relationship between these predictors and the probability of occurrence of *L. monopetalum*. The suitable ranges for the main contributing variables to the prediction of the species as was revealed from the response curve were as follows: 0.19–0.234 km for the distance from the coastline, 20.25–27.25 °C for the mean temperature of driest quarter (bio9); − 83.15–204.44 m for the suitable elevation for *L. monopetalum*, 33.74–141.2 mm for the precipitation of coldest quarter (bio19); 0.456–3.29 mm the precipitation of warmest quarter (bio18). The suitable habitats for *L. monopetalum* are the coastal sand dunes. The optimum range of the LST (Night) for the occurrence of the species was 293.06–311.02, and 293.06–311.02 for the LST (Day), whereas suitable range of NDVI was − 0.0223–0.5859, the NDWI − 0.080–0.332, NDDI 0.997–1.0011 and the EVI − 0.1424–0.4149. The probability of occurrence of the species exhibited a positive relationship with the elevation, bio18, bio19, NDWI, NDVI and EVI. In contrast, it exhibited a negative relationship with the distance to coastline, nitrogen, silt content, bio9, clay content, coarse fragments NDDI, LST (Day) and LST (Night).

### Predicted distribution of suitable areas using spectral indices

**Fig. 6:**
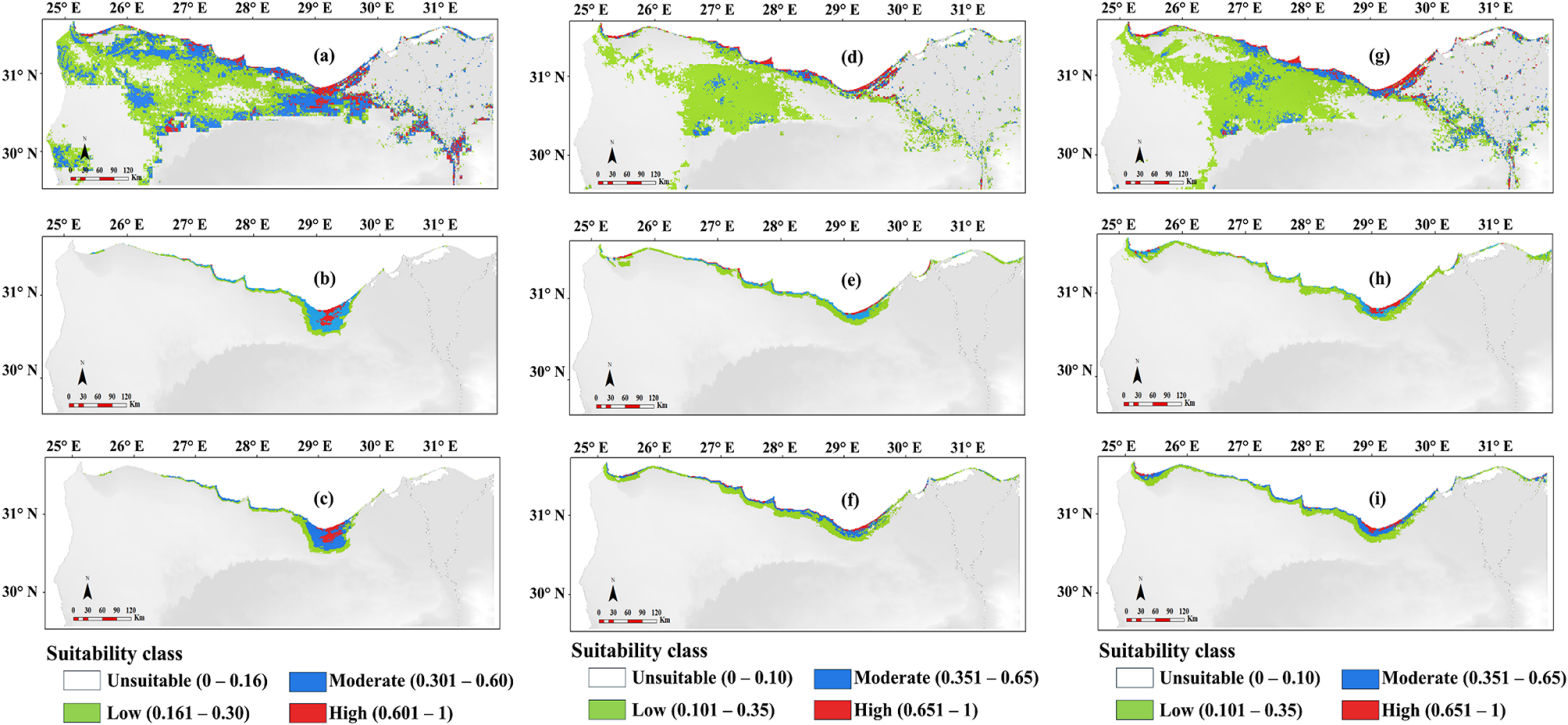
The predicted potential distribution of the studied species by Maxent model under current climate conditions, (**a**) *Thymelaea hirsuta* (RS-only), (**b**) *Thymelaea hirsuta* (EN-only), (**c**) *Thymelaea hirsuta* (CM), (**d**) *Ononis vaginalis* (RS-only), (**e**) *Ononis vaginalis* (EN-only), (**f**) *Ononis vaginalis* (CM), (**g**) *Limoniastrum monopetalum* (RS-only), (**h**) *Limoniastrum monopetalum* (EN-only) and (**i**) *Limoniastrum monopetalum* (CM). The map in figure was produced by the authors through processing of both remote sensing data and environmental data. The remote sensing MODIS satellite data were downloaded from https://earthexplorer.usgs.gov. The mean of the monthly values for each spectral indices for the period from 1/1/2021 to 1/1/2022 was calculated and used in modelling the distribution of the studied species. The environmental variables representing the current climatic conditions used for the construction of the species distribution model were acquired from the World Climate Database (Fick and Hijmans 2017; http://worldclim.org/version2). The bioclimatic data were downloaded from the WorldClim dataset version 2.1 at 30 arc-seconds (1 km) spatial resolution. More details on the data used in the analysis and production of the figures are included in the “Methods” sections.

The prediction of *T. hirsuta* distribution by Maxent model based on RS-only model showed good consistency with the existent distribution for the species (Fig. 6a). The total predicted suitability area was 32,597 km^2^ (18.62% of the total study area). The high, moderate, and low suitability areas are projected to cover 1189, 6266, 25,142 km^2^ (0.7, 3.6, 14.4% of the study area) respectively.

The predicted potential suitable area for the occurrence of *T. hirsuta* under current climate conditions based on EN-only model (Fig. 6b) in the western Mediterranean coastal region covered a total area of area 4829 km^2^ (2.76% of the total study area). The area predicted as of high suitability accounted for 11.43% of the total predicted suitable area, while the moderate, and the low suitability area attained 49.26 and 39.30% respectively.

The potential distribution of *T. hirsuta* based on integrating the combined model revealed that the low suitable areas will cover 4233 km^2^ (2.4% of the study area), while the moderate and high suitable areas will cover 2130 and 274 (1.4 and 0.2% % of the study area) respectively (Fig. 6c).

The prediction of *O. vaginalis* distribution by Maxent model based on the spectral indices alone showed good consistency with the existent distribution for the species (Fig. 6d). The total predicted suitability area was 32,597 km^2^ (18.62% of the total study area). The high, moderate, and low suitability areas are projected to cover 1034, 1795, 10,438 km^2^ (0.6, 1.0, 6.0% of the study area) respectively. The major suitability area (Moderate and highly suitable area) of *O. vaginalis* was located along the coastal line which is characterized by coastal sand dunes. The other suitable area (Low suitable area) was located far from the coastline.

The predicted suitable areas for the distribution of *O. vaginalis* were concentrated near the coast of the Mediterranean Sea, where the suitable habitat with the optimum temperature and precipitation conditions for the species prevail (Fig. 6e). The predicted potential suitable area for the occurrence of the species under current climate conditions based on EN-only model in the western Mediterranean coastal region covered a total area of 5534 km^2^ (3.16% of the total study area). The area predicted as of high suitability accounted for 412 km^2^ (7.44% 11.43% of the total precited suitable area), while the moderate, and the low suitability area attained 1279 (23.11%) and 3843 km^2^ (69.44%) respectively.

The potential distribution of *O. vaginalis* based on integrating the spectral indices with environment variables revealed that suitable areas for the species will cover 6411 km^2^ (3.66% of the total study area) (Fig. 6f). The low suitable areas will cover 3985 km^2^ (2.3% of the study area), while the moderate and high suitable areas will cover 1929 and 497 (1.1 and 0.3% % of the study area) respectively.

The prediction of *L. monopetalum* distribution by Maxent model based on the spectral indices alone showed good consistency with the existent distribution for the species (Fig. 6g). The total predicted suitability area was 15,941 km^2^ (9.11% of the total study area). The high, moderate, and low suitability areas are projected to cover 1149, 2975, 11,817 km^2^ (0.7, 3.6, 14.4% of the study area) respectively. Most probably moderate and highly suitable habitats were near and along the coastline of the Mediterranean Sea.

The predicted potential distribution of *L. monopetalum* under current climate conditions based on environmental predictors by using the Maxent model will occupy an area that represents 3.73% of total study area. Out of this area, the area predicted as of high suitability accounted for 6.86% of the total predicted suitable area, while the moderate, and the low suitability area attained 22.29% and 70.85% respectively (Fig. 6h).

The potential distribution of *L. monopetalum* based on integrating the spectral indices with environment variables revealed that the low suitable areas will cover 2.8% of the study area, while the moderate and highly suitable areas will cover 1.2 and 0.3% of the study area, respectively. The potential habitats with moderate and high suitability are distributed between the coastal areas to Alexandria to Sidi Abd El-Rahman and from Sidi Barrani to El-Sallum (Fig. 6i).

## Discussion and conclusion

The studied species—*Thymelaea hirsuta*,* Ononis vaginalis*, and *Limoniastrum monopetalum*—are Mediterranean endemics and key indicators of the primary habitats in the northwestern coastal deserts of Egypt. These species are increasingly threatened by population declines and the rapid loss and degradation of their natural habitats due to recent human activities^12,13,64^.

*Thymelaea hirsuta* is found across different sections of the Mediterranean phytogeographical region, including Mariut, Deltaic, and Sinai. Its distribution spans broad ecological gradients from the coast to the inland desert, primarily influenced by variations in aridity and the transition from calcareous to siliceous deposits^65^. The ongoing decline of this species in the western coastal desert has been linked to changes in precipitation and temperature regimes, including a decrease in annual rainfall and rising air temperatures. *T. hirsuta* is also recognized for its medicinal properties, with extensive research supporting its pharmacological applications. For example, El Amrani et al. reported its hypoglycemic and antidiabetic properties^32^, while Amari et al. highlighted its richness in natural antioxidants, suggesting its potential as a natural alternative to synthetic antioxidants^33^. Additionally, Felhi et al. identified antimicrobial activity in its essential oils^34^, and Dahamna et al. found that its extracts exhibit antifungal properties^35^.

*Ononis vaginalis* is primarily restricted to coastal dunes, where annual rainfall averages around 150 mm, but it becomes less common in inland desert areas^37^. Migahid et al. noted that low-salinity dunes provide an optimal microhabitat for this species^66^. Its specialization in coastal dune habitats suggests adaptations to environmental factors such as high soil carbonate content, low organic matter, alkalinity, and low total soluble salts^67^. However, *O. vaginalis* is also known to tolerate a range of soil moisture conditions^68^. Amer et al. isolated several flavonoids from *O. vaginalis*, including apigenin, chrysin, eupatilin, and astragalin^39^.

*Limoniastrum monopetalum* is highly adaptable to various environmental stresses, including salinity, intense radiation, water deficiency, high temperatures, and nutrient-poor soils^41,42^. These characteristics make it an ideal candidate for xeriscaping and landscape architecture in semi-arid Mediterranean regions. Ecologically, *L. monopetalum* plays a vital role in coastal dune stabilization, functioning as a sand accumulator, windbreaker^41^, and inhibitor of soil erosion. It also exhibits tolerance to oil-contaminated soils^43^ and has been identified as a promising species for the phytoremediation of heavy metal and petroleum hydrocarbon-polluted sites^44^. Additionally, its high nutritional value makes its vegetative parts suitable for fodder production^45^. Furthermore, *L. monopetalum* is rich in phenolic compounds and is regarded as a natural antioxidant source for human consumption. Mohammed et al. reported that its aqueous extract has a strong capacity for silver nanoparticle production^46^.

The AUC value is the dominant measure for assessing model performance, mostly for its insensitivity to the threshold selection^62,69–71^. However, high AUC, and TSS values do not always guarantee the accurate delineation of potential species distributions^19,72^. The measures of models’ evaluation of *T. hirsuta* indicated that the model which integrated the remotely sensed data with environmental variables exhibited slightly higher performance and accuracy than that based on the spectral indices alone and environmental alone. That suggests that the spectral indices used slightly improved the accuracy and performance of the model when combined with the environmental data. The excellent predictive capability of the model generated in the study was also further supported by a relatively high TSS value, which is a reliable measure of model evaluation^73^.

The prediction of the current distribution of *O. vaginalis* through models that combined environmental and spectral indices showed slightly higher performance and accuracy than the model that used spectral indices alone and environmental alone as indicated by all the measures of models’ evaluation.

The model predicting *L. monopetalum* distribution which integrated spectral indices with environmental also exhibited slightly higher performance and accuracy relative to that based on the other models. That suggests that the spectral indices used slightly improved the performance and the accuracy of the model when combined with the environmental data. However, the difference between the two models based on the AUC was limited but based on the TSS the model with combined predictors attained higher value. The result of the two models proved that combining environmental variables with remote sensing predictors increases the performance and accuracy of the models.

The models’ performance indicated that the combination of environmental variables and remotely sensed data performed slightly better than the use of the remotely sensed data alone and environmental predictors alone in predicting the distribution of the species. The integration of spectral indices such as NDVI and LST allows for capturing fine-scale habitat characteristics that are crucial for accurately predicting species distributions^15,17^. The consistent better performance of the models that used the combined environmental and remotely sensed data suggests that these two types of variables, when combined, improve the predictive power of the models. In contrast, models based solely on remote sensing data show the lower performances, even when compared with full models. The limited improvements detected have important implications for forecasting of the present species distribution. However, the statistical significance of the differences in model performance -comparing those based solely on environmental factors to those incorporating remotely sensed data-requires further assessment through rigorous statistical analyses. The inclusion of spectral indices may still contribute to refining model predictions in specific ecological contexts. Additionally, the slight improvement observed in the combined models may be due to the inherent correlation between spectral indices and bioclimatic variables. The bioclimatic variables in the WorldClim dataset were derived using thin-plate splines from weather station data, which were integrated with some remotely sensed data collected by MODIS^49^. Therefore, further similar comparative studies should explore climatic datasets that do not incorporate remotely sensed data.

Remote sensing has been one of the most powerful efficient approaches in terms of time and costs that provide observations useful for modelling distribution patterns of key species. The integration of different predictor sets in SDM approaches can strongly influence the precision and reliability of results. Commonly, specific numbers of variables are required to receive optimal predictions^74^ and different combinations of predictors may capture different parts of the target species’ niche^75,76^. The availability of multi-temporal satellite data at varied spatial resolutions is crucial for modeling the distribution of wild species^15,17^. Combining remote-sensing data with species distribution models, though still in their infancy, shows great promise for advancing theoretical and applied ecological research. This combination provides specific benefits: (1) expanding the range of environmental variables beyond topographic and climatic parameters, (2) enhancing the spatial resolution of input data from interpolated surfaces to direct geo-referenced measurements, and (3) incorporating seasonal information from multi-temporal remote-sensing imagery. Remote sensing products have been recognized for their transformative potential in providing simple and flexible distribution predictions^18,19^. Typically, an optimal number of variables are required for accurate predictions^74^, and different predictor combinations may capture various aspects of a species’ niche^75,76^. The advancement of in different analytical techniques, increased computational capacity, enhanced sensor fusion, and networking capabilities, along with free access to satellite data, have greatly promoted the use of remote sensing in species distribution modeling^77^.

In the present study, it was found that four (LST (Day), EVI, NDWI, and NDVI) out of the six utilized spectral indices contributed highly to the prediction of *T. hirsuta* current distribution based on RS-only model. The NDWI and LST (Day) were the best indices for predicting the current distribution of *T. hirsuta*. The NDWI is recognized as an efficient indicator of water stress, soil, and vegetation moisture conditions^58^. Moisture insufficiency can lead to physiological disturbances that may inhibit cell processes leading to the reduction in plant growth^78^. MODIS Land Surface Temperature (LST) data are increasingly utilized in species distribution models (SDMs) to understand and predict ecological processes^79,80^. Land Surface Temperature (LST) plays a critical role in controlling many physical, chemical, and biological processes^81,82^, and it correlates with soil moisture and canopy evapotranspiration^83,84^. By integrating environmental variables with spectral indices, both the Normalized Difference Water Index (NDWI) and LST (Day) emerge as key predictors in forecasting the potential distribution of species. Along with the distance to the coastline, bio8 (mean temperature of the wettest quarter), and bio9 (mean temperature of the driest quarter), these factors contribute significantly to the prediction models. The NDWI contributed highly to prediction of the current distribution of *O. vaginalis.* The current potential distribution of *O. vaginalis* is mainly controlled by how the species are located from the coastline, as areas near the coast receive a high percentage of precipitation. The *O. vaginalis* has a narrow ecological niche and it is a characteristic species for sand dunes habitat that is located near or on the shoreline.

The NDWI, NDVI and LST (Day) contributed highly to prediction of the current distribution of *L. monopetalum*. NDVI can help in detection of vegetation, monitoring and evaluating the stress levels, and the changes in vegetation due to human interferences, natural disturbances including climate change, wildfires, or changes in plants’ phenological stages. The potential current distribution of *L. monopetalum* shows that the species concentrated along the Mediterranean shoreline. The *L. monopetalum* species has a limited ecological niche compared to *T. hirsuta* that can be found in almost all types of habitats. So, *L. monopetalum* has a medium ecological niche. This result dovetails with the result of a combined model (environmental data with remote sensing data). By integrating environmental variables with remote sensing data, researchers can better understand the habitat requirements and potential distribution of threatened species, enabling more targeted conservation efforts^20^. Integration of spectral indices derived from remotely sensed data with other environmental variables are highly recommended for predicting the distribution of species native to Mediterranean ecosystems and similar ecosystems. Remotely sensed data (MODIS data) can improve the performance of SDMs by identifying habitat characteristics and environmental gradients that may exist among sites with similar climatic conditions.

The synergetic combination of remote-sensing data with environmental variables in species distribution models is still in its early stages but holds promising potential for new approaches in both theoretical and applied ecological research. Each dataset offers unique advantages, and their integration is expected to provide richer and more predictive information. This integrated approach can enhance the accuracy and reliability of predictions by leveraging the strengths of both remote-sensing and environmental data, thereby offering a more comprehensive understanding of species distributions. The combined models for *T. hirsuta*, *O. vaginalis*, and *L. monopetalum* consistently outperformed the models that utilized only environmental or remotely sensed data. Key environmental predictors such as the distance to coastline and climatic variables, alongside remote sensing indices like NDWI and LST, were crucial in determining the potential distribution of these species. These findings highlight the importance of a multifaceted approach in species distribution modeling, which can improve conservation planning and management strategies for native Mediterranean plant species in the face of changing environmental conditions. Integrating various data types enables a more accurate and comprehensive understanding of species distribution, facilitating better-informed conservation efforts. The study revealed the enhanced predictive accuracy achieved by integrating remotely sensed data with environmental variables. This integration not only improves the spatial distribution predictions of species but also supports more effective conservation planning and management strategies. As the Mediterranean region faces increasing environmental pressures, the outcomes of this research underscore the importance of incorporating advanced remote sensing techniques in biodiversity conservation efforts.

## Electronic supplementary material

Below is the link to the electronic supplementary material.

Supplementary Material 1

## Data Availability

The datasets generated during and/or analyzed during the current study are available from the corresponding author on reasonable request.
