# Supplementary material for: Remotely sensed data contribution in predicting the distribution of native Mediterranean species
Source: Sci Rep. 2025 Apr 11;15:12475. doi: 10.1038/s41598-025-94569-y (PMC11992134; doi:10.1038/s41598-025-94569-y)
Supplement: Supplementary file 1 — Supplementary Material 1 [file 41598_2025_94569_MOESM1_ESM.docx]

**Appendices**


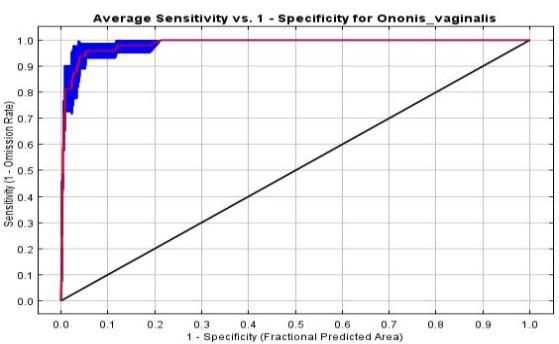

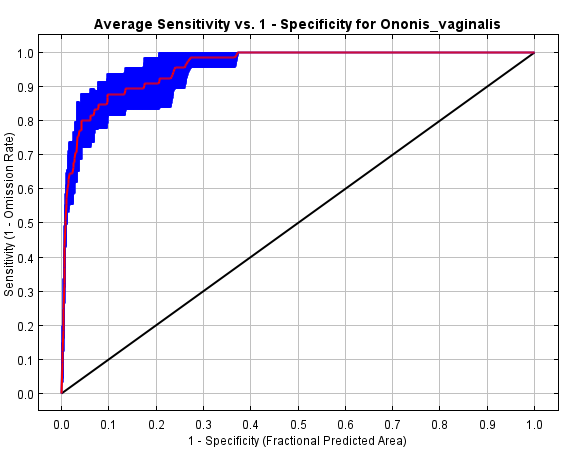

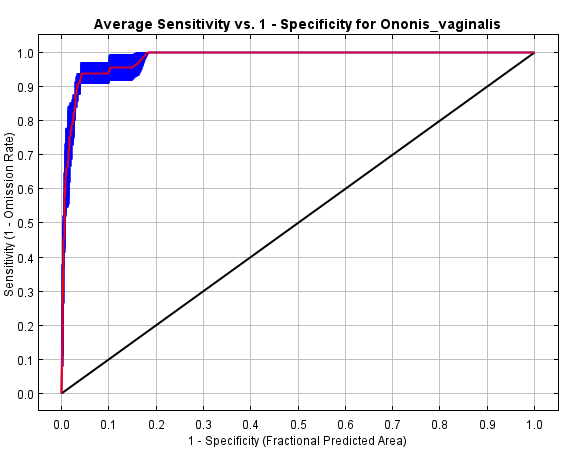


(a)


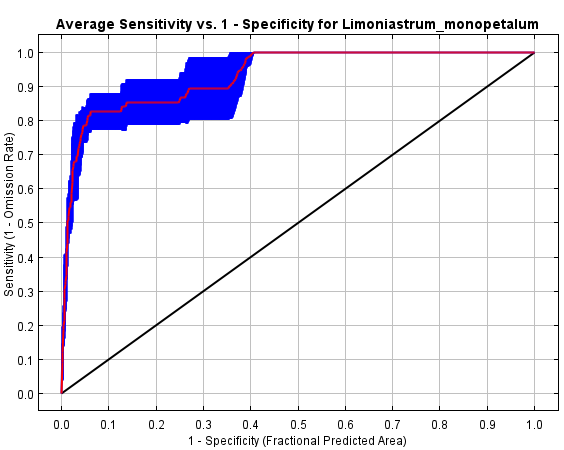

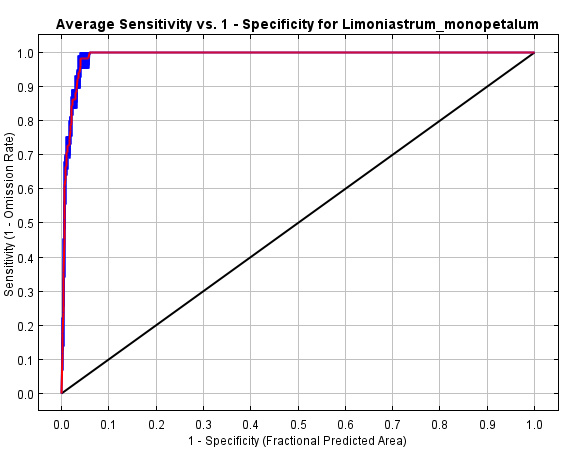

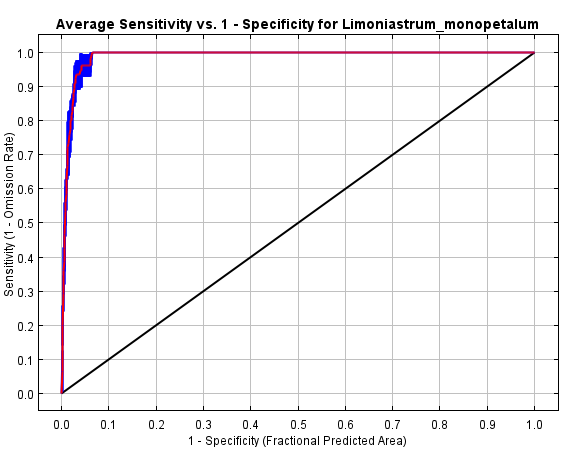

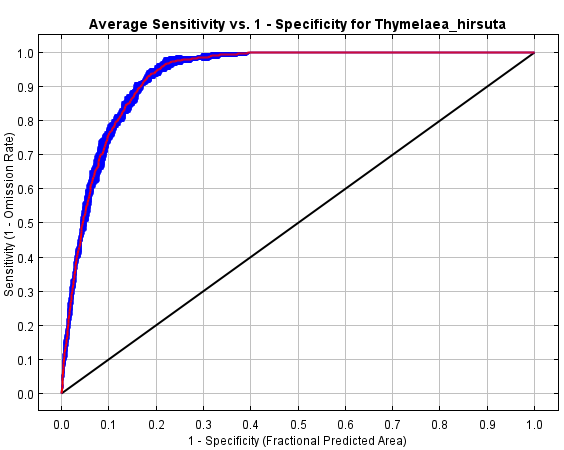


(a)


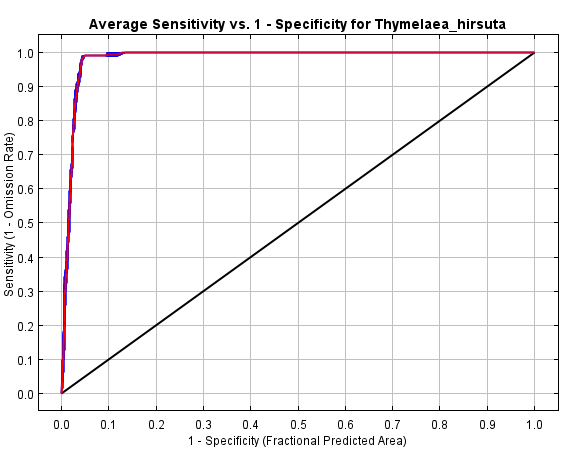

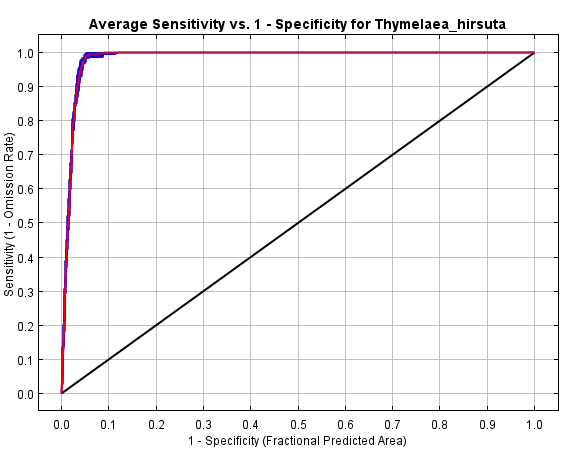


Mean (AUC = 0.931)

Mean +/- one stddev

Random Prediction


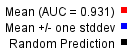


Mean (AUC = 0.983)

Mean +/- one stddev

Random Prediction


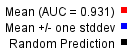


Mean (AUC = 0.99)

Mean +/- one stddev

Random Prediction


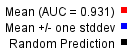


Mean (AUC = 0.95)

Mean +/- one stddev

Random Prediction


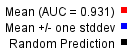


Mean (AUC = 0.980)

Mean +/- one stddev

Random Prediction


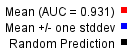


Mean (AUC = 0.985)

Mean +/- one stddev

Random Prediction


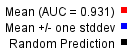


Mean (AUC = 0.933)

Mean +/- one stddev

Random Prediction


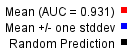


Mean (AUC = 0.98)

Mean +/- one stddev

Random Prediction


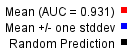


Mean (AUC = 0.99)

Mean +/- one stddev

Random Prediction


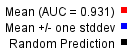


(g)

(h)

(i)

(d)

(e)

(f)

(a)

(b)

(c)

**Fig. S1.** The receiver operating characteristics (ROC) curve of the Maxent model predicts the potential distribution of the studied species; (a) *Thymelaea hirsuta* (RS-only) (b) *Thymelaea hirsuta* (EN-only), (c) *Thymelaea hirsuta*  (CM), (d) *Ononis vaginalis* (RS-only) (e) *Ononis vaginalis* (EN-only), (f) *Ononis vaginalis* (CM), (g) *Limoniastrum monopetalum* (RS-only), (h) *Limoniastrum monopetalum* (EN-only) and (i) *Limoniastrum monopetalum* (CM). under current climate conditions. The red curve shows the mean response, and the blue margins are +/− one standard deviation calculated over 5 replicates.


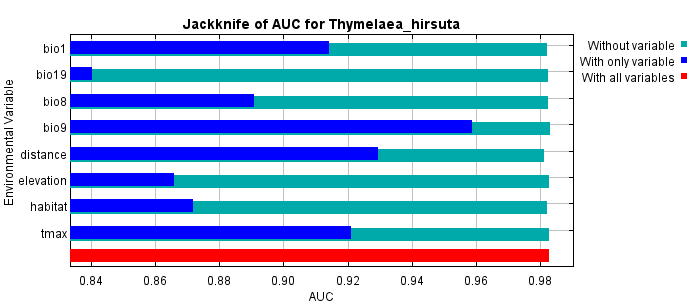

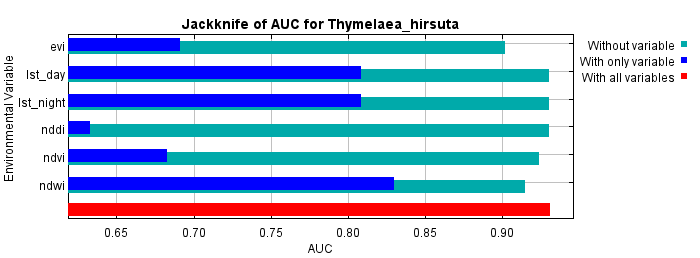

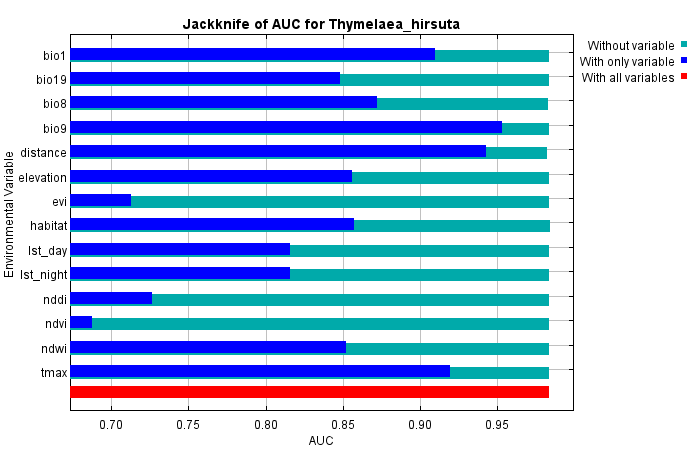

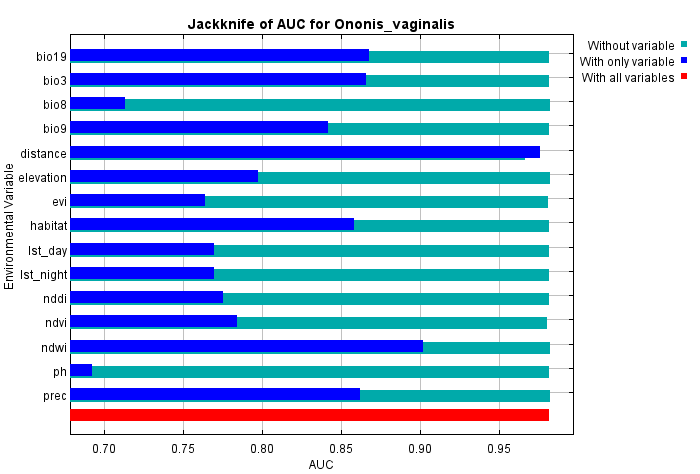

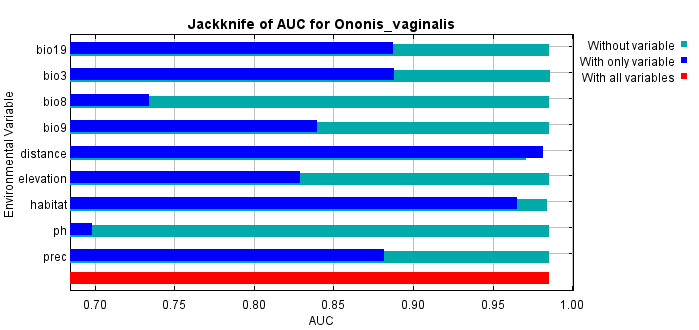

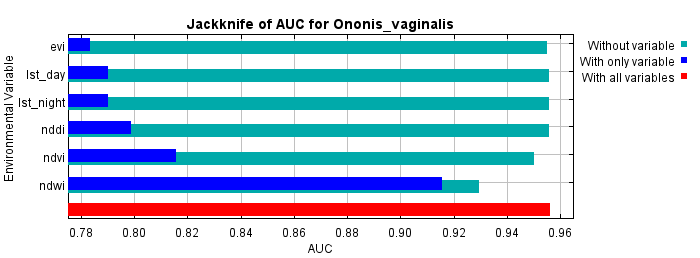

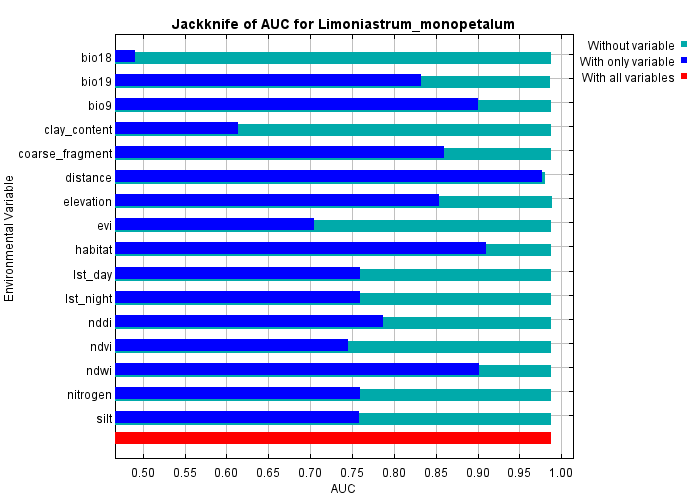

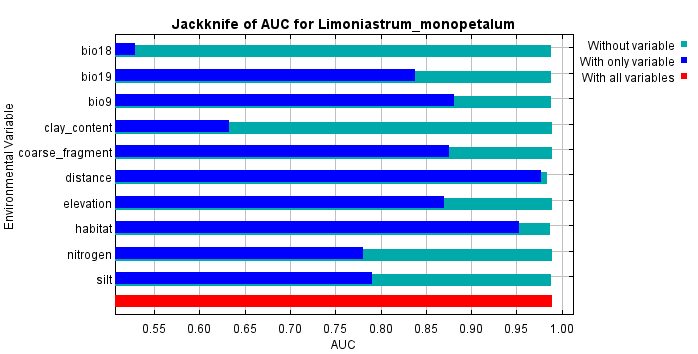

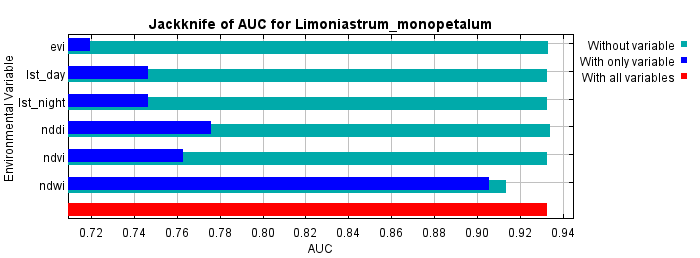


(a)

(b)

(c)

(g)

(h)

(i)

(d)

(e)

(f)

**Fig. S2.** The Jackknife test of variable importance using AUC on test data for the Maxent model predicting the distribution of the studied species; (a) *Thymelaea hirsuta* (RS-only) (b) *Thymelaea hirsuta* (EN-only), (c) *Thymelaea hirsuta*  (CM), (d) *Ononis vaginalis* (RS-only) (e) *Ononis vaginalis* (EN-only), (f) *Ononis vaginalis* (CM) , (g) *Limoniastrum monopetalum* (RS-only), (h) *Limoniastrum monopetalum* (EN-only) and (i) *Limoniastrum monopetalum* (CM).
